# Long-Term Changes in the Biomarkers of Left Atrial Fibrosis after Pulmonary Vein Isolation for Paroxysmal and Persistent Atrial Fibrillation

**DOI:** 10.31083/j.rcm2406171

**Published:** 2023-06-12

**Authors:** Lilla Szuromi, Orsolya Hajas, Edina Nagy-Baló, Ildikó N. Forgács, László T. Nagy, Miklós Fagyas, Attila Tóth, Béla Nagy Jr, János Kappelmayer, Zoltán Csanádi

**Affiliations:** ^1^Division of Cardiology, Department of Cardiology, Faculty of Medicine, University of Debrecen, H-4032 Debrecen, Hungary; ^2^Division of Clinical Physiology, Department of Cardiology, Faculty of Medicine, University of Debrecen, H-4032 Debrecen, Hungary; ^3^Department of Laboratory Medicine, Faculty of Medicine, University of Debrecen, H-4032 Debrecen, Hungary

**Keywords:** pulmonary vein isolation, atrial fibrillation recurrence, fibrosis biomarkers, atrial diameter

## Abstract

**Background:**

Atrial fibrillation (AF) is accompanied by inflammation and fibrosis to variable extent. The biomarkers of fibrosis were measured in patients with different forms of AF and cardiac status. Herein, we assessed the associations of the baseline concentrations of different biomarkers with the long-term success of pulmonary vein isolation (PVI) in patients with a structurally normal heart. Furthermore, we compared biomarker levels before and 3 years after ablation to gain further insights into the AF mechanism.

**Methods:**

Patients, undergoing PVI for paroxysmal/persistent AF were enrolled prospectively. Blood samples were obtained 24 hours before and 3 years after ablation. Serum cancer antigen 125 (CA-125), plasma Caspase-3, Galectin-3 and Cathepsin L concentrations were measured. Follow-up visits every 6 months included 12-lead electrocardiogram, 24-hour Holter, trans-telephonic monitoring as well as transthoracic echocardiography after ablation. Biomarker levels, left ventricular ejection fraction and left atrial (LA) diameters at baseline and at the 3-year follow-up were compared in patients with versus without AF recurrence.

**Results:**

A total of 63 patients were enrolled (23 women; age 61.4 (± 8.8) years). The acute isolation of all pulmonary veins was achieved in all patients. During a mean follow-up of 36.3 ± 6.3 months, AF recurrence was demonstrated in 26 (41.3%) patients. No significant differences were demonstrated in the levels of CA-125, Galectin-3, Caspase-3 and Cathepsin L pre- and post-ablation in patients with versus without AF recurrence. A significant decrease was detected in the concentrations of Caspase-3, Galectin-3 and Cathepsin L during follow-up with no difference in patients with versus without AF recurrence. A positive correlation was found between Caspase-3 levels and LA diameters in the AF recurrence group both before (r = 0.477; *p *= 0.018) and after the procedure (r = 0.533; *p *= 0.019).

**Conclusions:**

Our results demonstrated that the levels of CA-125, Caspase-3, Cathepsin L and Galectin-3 are not associated with AF recurrence after PVI in patients with a structurally normal heart and mainly paroxysmal AF. Except for CA-125, all the other biomarkers demonstrated a significant decrease during a 3-year follow-up post-ablation. Furthermore, Caspase-3 levels demonstrated a positive correlation with LA dimensions in patients with AF recurrence.

## 1. Introduction

Pulmonary veins (PVs) play an essential role in the pathomechanism of atrial fibrillation (AF) as known predilection sites for myocardial sleeves, also capable of rapid electrical firing, which may trigger or sustain the arrhythmia [1]. Catheter ablation with the electrical isolation of all PVs is an established method to maintain sinus rhythm (SR) in many patients, especially in younger ones with a structurally normal heart and no significant comorbidities [2]. Pulmonary vein isolation (PVI) is associated with a high success rate in this patient cohort, and long-term failure is often the result of the electrical reconnection of one or more PVs after an acute success of PVI. However, in some of the younger patients with a structurally normal heart and paroxysmal AF (referred to as “lone” AF according to earlier terminology) and more common in the elderly with significant cardiac and extracardiac comorbidities and persistent or long-term persistent forms of the arrhythmia, the role of other mechanisms related mostly to the fibrosis of the atrial myocardium might become more dominant and thus less benefit can be expected from the sole electrical isolation of the PVs [3].

Diagnostic methods to assess the left atrial (LA) substrate include the measurement of LA volume with echocardiography and the evaluation of fibrosis with cardiac magnetic resonance imaging (CMRI) by using gadolinium late enhancement technique [4]. Moreover, in pre-clinical as well as in human studies the plasma or serum levels of different biomarkers of myocardium fibrosis and inflammation have been compared in patients with and without different forms of AF [5, 6, 7, 8]. These studies investigated the levels of Cancer antigen 125 (CA-125), Caspase-3, and Cathepsin L in different patient cohorts, including patients mostly with persistent and long-standing persistent forms of AF and with significant comorbidities, such as chronic heart failure (HF). Limited data are also available on the relationship between pre-ablation levels of Galectin-3 and AF recurrence during the 1-year follow-up after ablation [9, 10, 11, 12, 13]. Although the indication for AF ablation has recently been extended to include patients with significant comorbidities, mainly congestive HF and those with persistent forms of arrhythmia, this procedure is still mostly offered to patients with shorter AF duration, a structurally normal heart, including LA size close to normal and limited comorbidities [2]. No sufficient data are available in this patient cohort regarding either the association of these biomarkers with the long-term success of PVI or the changes in the levels of these substances.

Herein, we report the results of our prospective clinical study on the levels of CA-125, Caspase-3, Galectin-3 and Cathepsin L obtained before PVI and after a minimum of a 3-year follow-up in patients with paroxysmal/persistent (excluding long-standing form) AF with a structurally normal heart. The aim of this research was to assess the association of these biomarkers with the long-term success of PVI in this patient cohort. Additionally, we evaluated long-term changes in the plasma/serum levels of these substances to gain further insights into the effects of AF ablation.

## 2. Materials and Methods

### 2.1 Patients

Patients undergoing PVI for documented paroxysmal/persistent AF at our Department were considered prospectively. The inclusion criteria were the following: (1) age 18–75 years, (2) failure of at least one antiarrhythmic drug and (3) willingness to sign a written informed consent. Exclusion criteria included: (1) long-standing persistent or permanent AF, (2) reversible cause of AF (e.g., hyperthyroidism), (3) presence of a LA thrombus, (4) previous heart surgery, (5) valvular heart disease, (6) left ventricular ejection fraction (LVEF) <50%, and clinical signs of HF, (7) unstable angina or myocardial infarction within the last 3 months, (8) acute inflammatory disorders, (9) pregnancy, (10) malignant disease and (11) previous AF ablation. The study design was in accordance with the guiding principles of the Declaration of Helsinki and was approved by the Regional and Institutional Ethics Committee of the University of Debrecen (DE RKEB/IKEB/4951-2018) and the National Institute of Pharmacy and Nutrition (OGYÉI/12743/2018). All patients signed a written informed consent form prior to inclusion.

### 2.2 Baseline Cardiac Evaluation

All patients were subjected to a full baseline evaluation, including medical history, physical examination, 12-lead electrocardiogram (ECG), transthoracic echocardiographic (TTE) examination to measure LVEF and LA diameters before the procedure. Transoesophageal echocardiography (TEE) was performed in all patients within 24 hours prior to the procedure in order to exclude the presence of a cardiac thrombus. Blood samples for the measurement of biomarkers were taken from a peripheral vein within 24 hours prior to the procedure.

### 2.3 Ablation Procedure

The procedures were performed under conscious sedation. The Seldinger technique was used for femoral vein cannulation. Multipolar electrode catheters were placed in the coronary sinus and in the right ventricle. A transseptal puncture was performed with a Brockenbrough needle under intracardiac echocardiography (ICE) guidance. After the transseptal puncture, 150-IU/kg body weight intravenous heparin bolus was given followed by continuous infusion to keep the Activated Clotting Time (ACT) level between 300–400 msec. All PVI procedures were performed with any of the following 3 ablation technologies at the operator’s discretion: (1) phased radiofrequency ablation (RFA) with the 2nd generation Pulmonary Vein Ablation Catheter (PVAC) Gold catheter (Medtronic Inc, 990078, Minneapolis, MN, USA); (2) cryoablation with the Arctic Front Advance catheter (Medtronic Inc, 2AF283, Minneapolis, MN, USA); (3) point-by-point PVI with focal irrigated RFA with contact force monitoring using a Thermocool, Smarttouch catheter (D133602, Biosense Webster Inc., Johnson & Johnson Medtech, Irvine, CA, USA). The procedural endpoint was the isolation of all PVs, which was verified with pacing manoeuvres. If necessary, SR was restored by cardioversion after the procedure. Each ablation protocol applied at our center is described in detail below [14, 15, 16].

#### 2.3.1 PVAC Ablation

The Mullins sheath was exchanged for a 12-Fr long FlexCath sheath and placed in the LA over a guidewire. The second-generation circular PVAC-Gold catheter (Medtronic Inc, 990078, Minneapolis, MN, USA), which contains 9 electrodes of gold alloy, was loaded into the introducer. Heparinized saline was flushed continuously to the sheath to minimize air ingress. RFA was performed by the targeted ablation of each PV-LA antrum in a temperature-controlled and power-limited manner (60 °C, maximum 10 W). The typical duration of each ablation session was 60 s.

#### 2.3.2 Cryoballoon Ablation

The Mullins sheath was exchanged for a 12-Fr long FlexCath sheath and placed in the LA over a guidewire. The second-generation Arctic Front Advance balloon was used in all cases. PV electrograms were monitored continuously via a circular electrode catheter (Achieve Mapping Catheter, 990063-020, Medtronic Inc, Minneapolis, MN, USA) placed in the PV during each freezing cycle. The balloon position in the PVs was assessed with the administration of contrast injection before each energy application. Durations of the freezing cycles were based on the achieved temperature and time to complete PVI. The target freezing temperature was between –40 °C and –55 °C.

#### 2.3.3. Point-by-Point Pulmonary Vein Isolation

A circular decapolar Lasso catheter was advanced to the ostium of each PV through the Mullins transseptal sheath. A 9F steerable Agilis sheath was also placed in the LA after a 2nd transseptal puncture, and a contact force ablation catheter was advanced to the LA. Point-by-point RFA was performed in a temperature-controlled and power-limited fashion guided by 3D electro-anatomical mapping. The typical duration was 30 s for each radiofrequency (RF) application. PVI was assessed based on intracardiac signals recorded through the electrodes of the Lasso catheter.

### 2.4 Laboratory Measurements of Biomarkers

Baseline blood samples were collected from the cubital vein within 24 hours prior to the procedure. Follow-up blood samples were collected from the cubital vein after the end of the 3-year follow-up period. Blood samples were collected into vacutainer tubes. Tubes containing ethylenediamine tetraacetic acid (EDTA) anticoagulant (3 mL Vacuette tube with K3EDTA, 454086, Greiner Bio-One GmbH Bad Haller Str. Kremsmünster, Austria) and tubes containing clot activator (serum tubes with polymer gel separator, 5 mL BD Vacutainer SST II Advance Plus Blood Collection Tubes, 367955, Becton, Dickinson and Company Franklin Lakes, NJ, USA) were used. Within two hours, the samples were centrifuged at 1500 *g* for 20 minutes at room temperature. From EDTA tubes the plasma phase was pipetted, from serum separator tubes the serum phase was pipetted into aliquots and these were stored at –70 °C until the analysis.

Serum CA-125 levels were determined by an electro-chemiluminescent microparticle immunoassay (Cobas® e602) (Roche Diagnostics, Mannheim, Germany). Plasma Caspase-3 concentration was measured with Human Caspase 3 ELISA kit (Thermo Fisher Scientific Inc., Carlsbad, CA, USA, Cat. No. BMS2012INST), while plasma Galectin-3 was measured with Human Galectin-3 DuoSet ELSA kit (R&D Systems, Inc., Minneapolis, MN, USA, Cat. No. DY1154) and plasma Cathepsin L with Human Cathepsin L DuoSet ELISA kit (R&D Systems, Inc., Minneapolis, MN, USA, Cat. No. DY952) according to the manufacturer’s instructions.

### 2.5 Post-Ablation Follow-Up

The previously prescribed antiarrhythmic drugs were continued after the procedure and stopped 3 months later. All patients were anticoagulated for a minimum of 3 months period after the procedure, then the decision on long-term treatment was based on the ${\mathrm{CHA}}_{2}$${\mathrm{DS}}_{2}$-VASc score. All patients had follow-up visits every 6 months post-ablation, which included clinical evaluation, 12-lead ECG and 24-hour Holter recordings. Patients were encouraged to have ECG recordings in case of any symptom suggestive of an AF episode. In these cases, patients were also offered trans-telephonic monitoring for up to 6 weeks to facilitate the documentation of the rhythm during palpitation. Recurrence was defined as any documented AF episode lasting more than 30 seconds. At the 3-year follow-up LVEF and LA diameter was measured with TTE examination.

### 2.6 Statistical Analysis

Statistical analysis was performed using GraphPad Prism version 8.0.0 for Windows (GraphPad Software, San Diego, CA, USA). Normality of data distribution was assessed with Kolmogorov-Smirnov test. Continuous variables are expressed as means ± standard deviation (SD) and variables not normally distributed as medians and interquartile range. Categorical data were presented as counts with percentages within brackets. Analyses were calculated using Student’s *t* test or the Mann–Whitney test for continuous variables. To compare baseline and follow-up measurements, a paired *t*-test or Wilcoxon test was applied. The differences between categorical variables were assessed by the Chi-square or Fisher’s exact test. Evaluation of correlation was analysed using the Spearman’s correlation coefficient. *p <* 0.05 was considered statistically significant.

## 3. Results

### 3.1 Baseline Patient and Procedure Characteristics

A total of 63 patients were included in the study (23 women; age 61.4 ± 8.8 years). Thirty-six (57.1%) patients had been on antiarrhythmic drug therapy before the procedure. PVI was performed with PVAC ablation in 11, with cryoballoon in 28, and with point-by-point focal irrigated RFA in 24 patients. The acute isolation of all PVs was achieved in all patients. There were no complications during the procedure. The baseline characteristics are presented in Table 1.

**Table 1.** **Baseline characteristics of patients with or without AF recurrence during the 3-year follow-up period**.

Characteristics	No recurrence (n = 37)	AF recurrence (n = 26)	*p*-value
Age (years)	56.5 ± 9.2	59.9 ± 8.5	0.158
Female sex	9 (24.3%)	14 (53.8%)	0.017
Paroxysmal AF	34 (91.9%)	20 (76.9%)	0.095
Persistent AF	3 (8.1%)	6 (23.1%)	0.095
${\mathrm{CHA}}_{2}$${\mathrm{DS}}_{2}$-VASc score	1.4 ± 1.2	1.8 ± 1.1	0.155
Ablation technique			
RFA	16 (43.2%)	8 (30.8%)	0.446
PVAC	7 (18.9%)	4 (15.3%)	
CRYO	14 (37.8%)	14 (53.8%)	
Left atrium (mm)	41.8 ± 4.1	41.3 ± 5.2	0.674
LVEF (%)	58.5 ± 5.3	57.0 ± 7.7	0.390

Normally distributed continuous data are presented as means with standard deviation and differences examined with Student’s *t*-test. Categorical data are presented as counts with percent values within brackets and tested with Chi-square or Fisher’s exact test. AF, atrial fibrillation; ${\mathrm{CHA}}_{2}$${\mathrm{DS}}_{2}$-VAS score based on the presence of congestive heart failure, hypertension, age ≥75, diabetes, stroke, vascular disease, age ≥65, sex; RFA, point-by-point radiofrequency ablation; PVAC, pulmonary vein ablation catheter; CRYO, cryoballoon ablation; LVEF, left ventricular ejection fraction.

### 3.2 Arrhythmia Recurrence during Follow-Up

During a mean follow-up of 36.3 ± 6.3 months, AF recurrence was demonstrated in 26 (41.3%) patients. 23% of the patients with recurrence had persistent AF at baseline as compared to 8% in the no recurrence group. The only statistical difference between the groups with or without recurrence was the higher proportion of female patients with AF recurrence (Table 1). All patients with recurrence had paroxysmal AF after the ablation.

### 3.3 Biomarker Levels at Baseline and 3 Years after Ablation

**Fig. 1.**
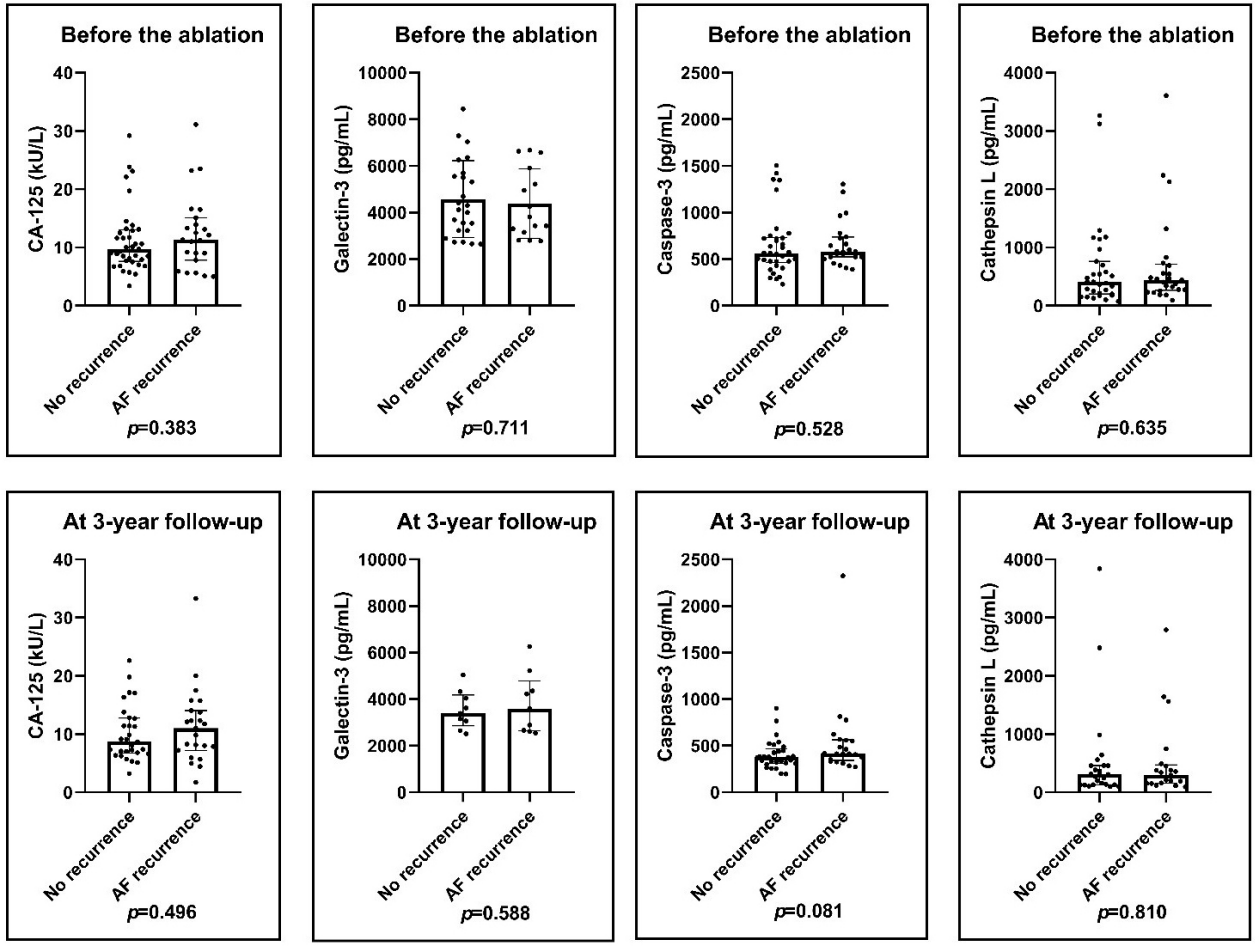
**Pre-ablation and post-ablation levels of the biomarkers**. There was no significant difference in CA-125, Galectin-3, Caspase-3 and Cathepsin L pre-ablation and post-ablation levels in patients with or without AF recurrence. Results of Mann–Whitney U-test. AF, atrial fibrillation; CA-125, cancer antigen 125.

Baseline (pre-ablation) biomarker levels in patients free of arrhythmia versus in those with AF recurrence during follow-up were compared. No significant differences in CA-125, Galectin-3, Caspase-3 and Cathepsin L levels were demonstrated between the two groups. Similarly, post-ablation blood samples taken 36.3 ± 6.3 months after the ablation procedure demonstrated no differences between the 2 groups in the level of any of the 4 substances measured (Fig. 1).

**Fig. 2.**
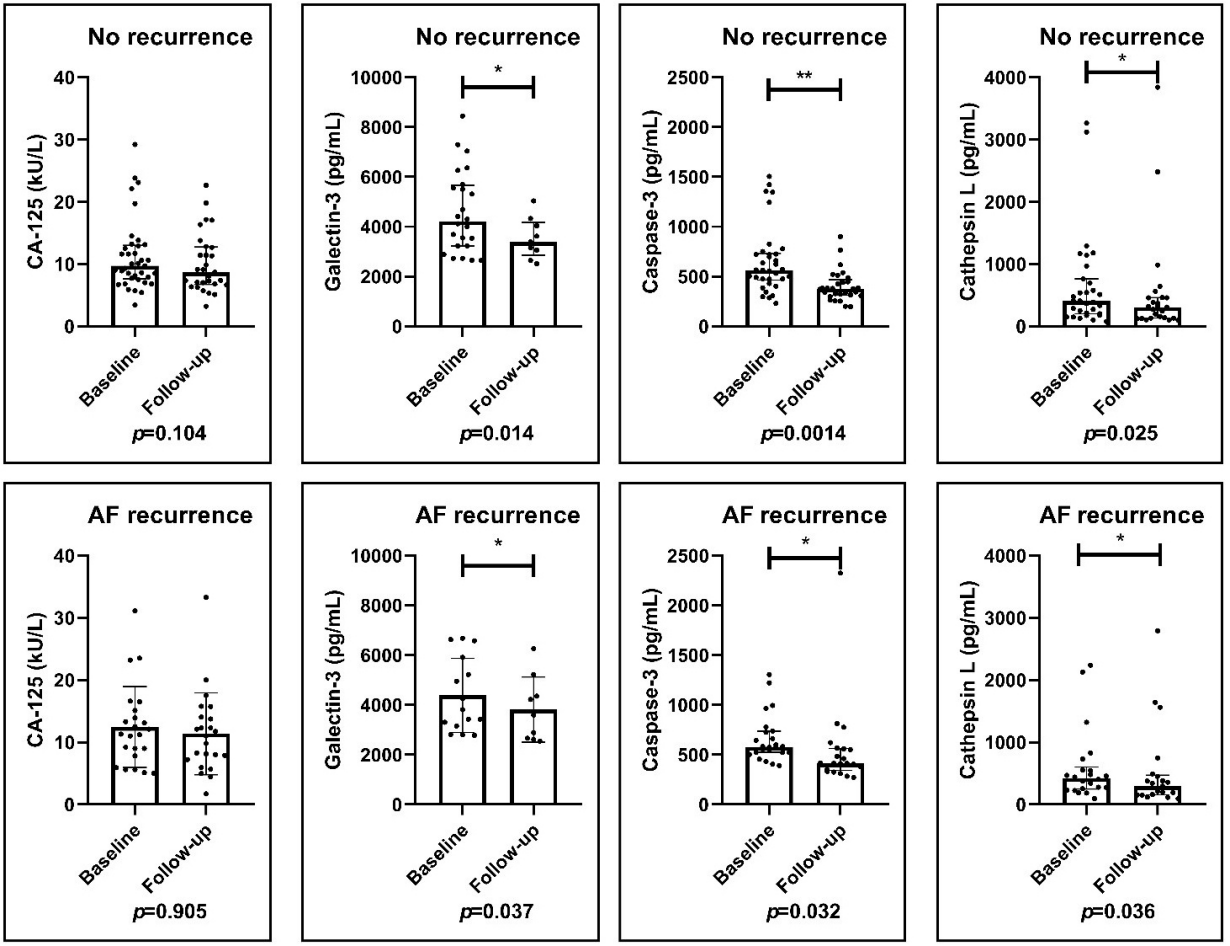
**Changes in biomarker levels before and after ablation**. The serum CA-125 levels were not changed significantly during the follow-up period in either group. The plasma Galectin-3, Caspase-3 and Cathepsin L levels decreased significantly both in the arrhythmia-free and the AF recurrence group. Results of paired *t*-test and Wilcoxon test. *p < 0.05; **p < 0.01. CA-125, cancer antigen 125; AF, atrial fibrillation.

Biomarker levels obtained at baseline were also compared to those obtained at the end of follow-up after PVI in both groups. Serum CA-125 levels showed no significant change neither in the no AF recurrence group (9.7 (7.6–13.0) kU/L vs 8.7 (6.7–12.8) kU/L; *p *= 0.104) nor in the recurrence group (11.3 (7.8–15.1) kU/L vs 11.0 (7.2–14.0) kU/L; *p *= 0.681). The plasma Galectin-3 levels decreased significantly both in the arrhythmia free (4209.9 (3226.2–5662.4) pg/mL vs 3391.4 (2856.2–4182.5) pg/mL; *p *= 0.014) and in the AF recurrence group (3810.4 pg/mL (3156.8–5915.8) vs 3591.9 (2637.8–4786.8) pg/mL; *p *= 0.037). Similarly, plasma Caspase-3 levels decreased significantly during the follow-up period in both groups (564.1 (463.0–731.6) pg/mL vs 376.9 (310.9–467.6) pg/mL;* p *= 0.001) and (579.0 (523.9–735.6) pg/mL vs 413.5 (341.8–563.5) pg/mL; *p *= 0.032) as well as plasma Cathepsin L levels decreased in the no recurrence group (406.9 (200.4–763.3) pg/mL vs 309.2 (131.7–460.4) pg/mL; *p *= 0.025) and in the recurrence group (422.4 (248.3–600.4) pg/mL vs 294.4 (158.2–470.7) pg/mL; *p *= 0.036) (Fig. 2).

### 3.4 Echocardiographic Parameters

**Fig. 3.**
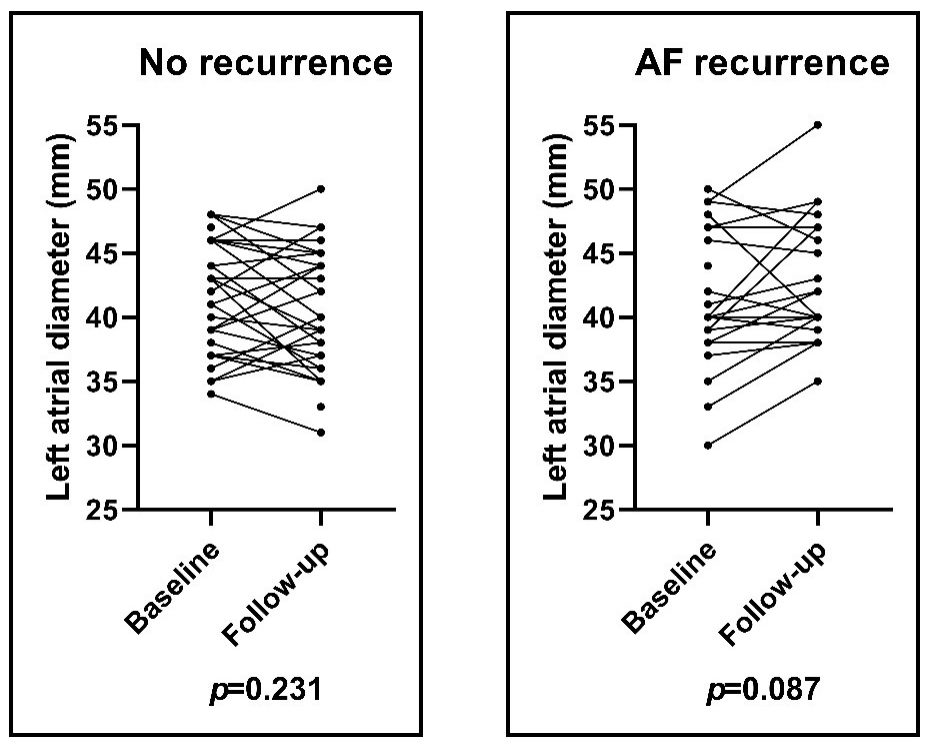
**Left atrial diameter changing during the 3-year follow-up period in patients with and without AF recurrence**. LA diameters did not change significantly in either group. Results of paired *t*-test. AF, atrial fibrillation; LA, left atrial.

LA diameter demonstrated a slight decrease from baseline to the end of follow-up in patients with no recurrence (41.8 ± 4.1 mm vs 40.5 ± 4.6 mm; *p *= 0.231) and a minimal increase in patients with AF recurrence (41.3 ± 5.2 mm vs 42.7 ± 4.9 mm; *p *= 0.087), however, none of these changes reached the level of statistical significance (Fig. 3). The LVEF was not changed significantly in the two groups (58.49 ± 5.3% vs 57.16 ± 4.3%; *p = *0.248; no recurrence group) (57.04 ± 7.7% vs 56.5 ± 5.5%; *p = *0.786; AF recurrence group).

### 3.5 Correlation of Biomarkers with Left Atrial Diameters

**Fig. 4.**
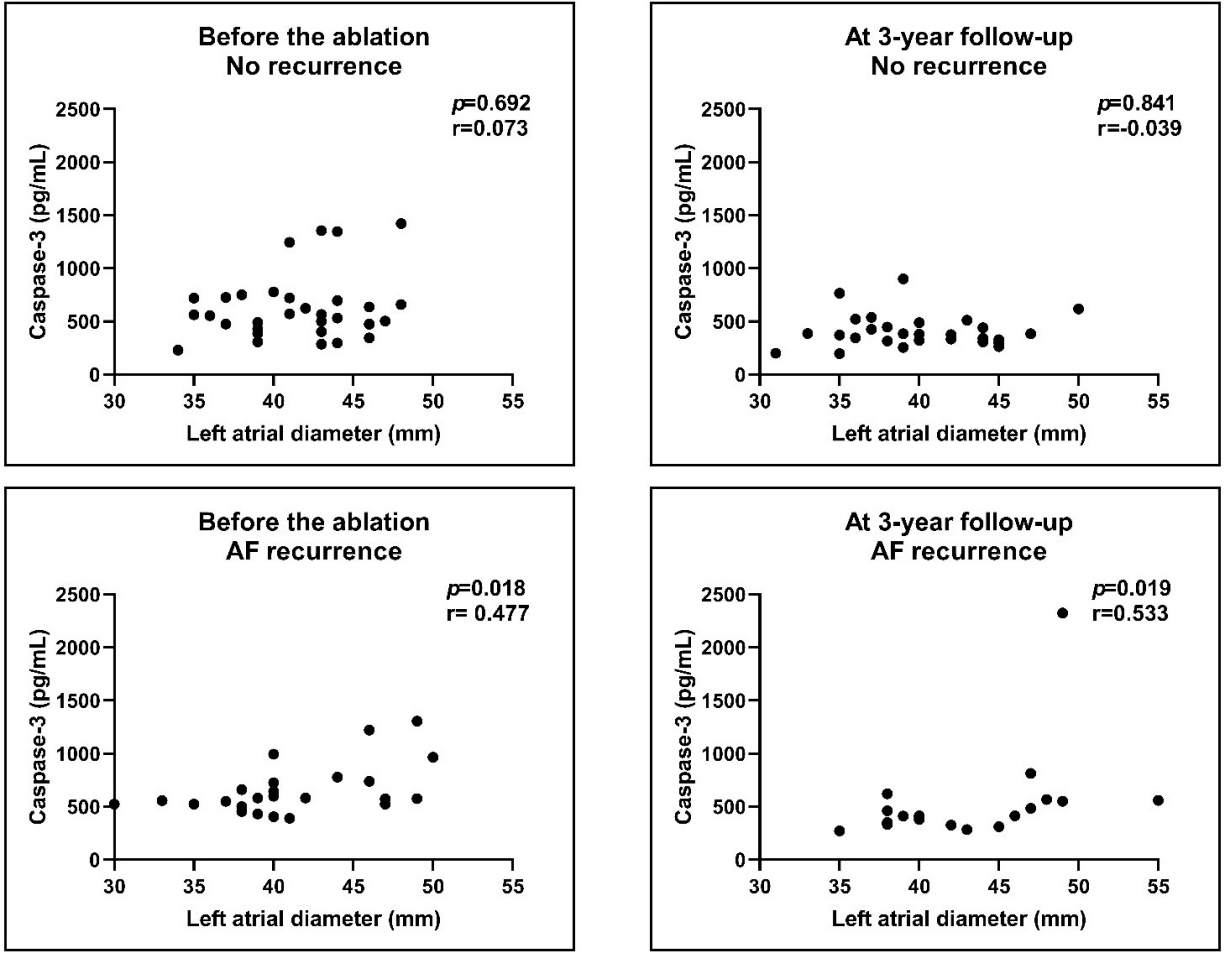
**Correlation of Caspase-3 levels with LA diameters before and after the procedure**. We compared the no recurrence group with the AF recurrence group. We found correlation between the Caspase-3 levels and LA diameters in the AF recurrence group before and after the procedure. There was no correlation in the arrhythmia-free group. Results of Spearman’s correlations. AF, atrial fibrillation; LA, left atrial.

**Fig. 5.**
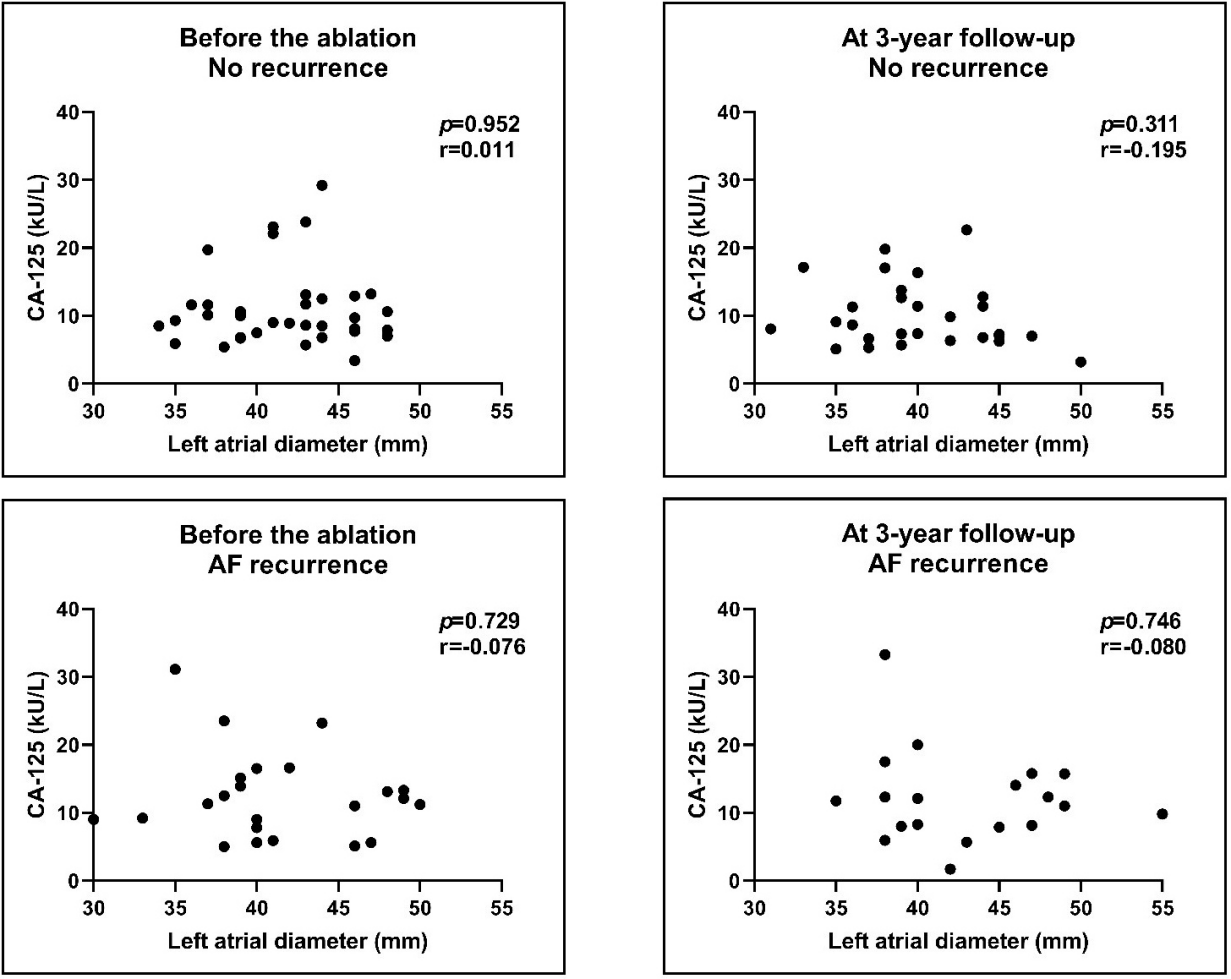
**Correlation of CA-125 levels with LA diameters before and after the procedure**. We compared the no recurrence group with the AF recurrence group. There was no correlation between the pre-ablation and post-ablation levels of CA-125 with the LA diameters. Results of Spearman’s correlations. AF, atrial fibrillation; LA, left atrial; CA-125, cancer antigen 125.

**Fig. 6.**
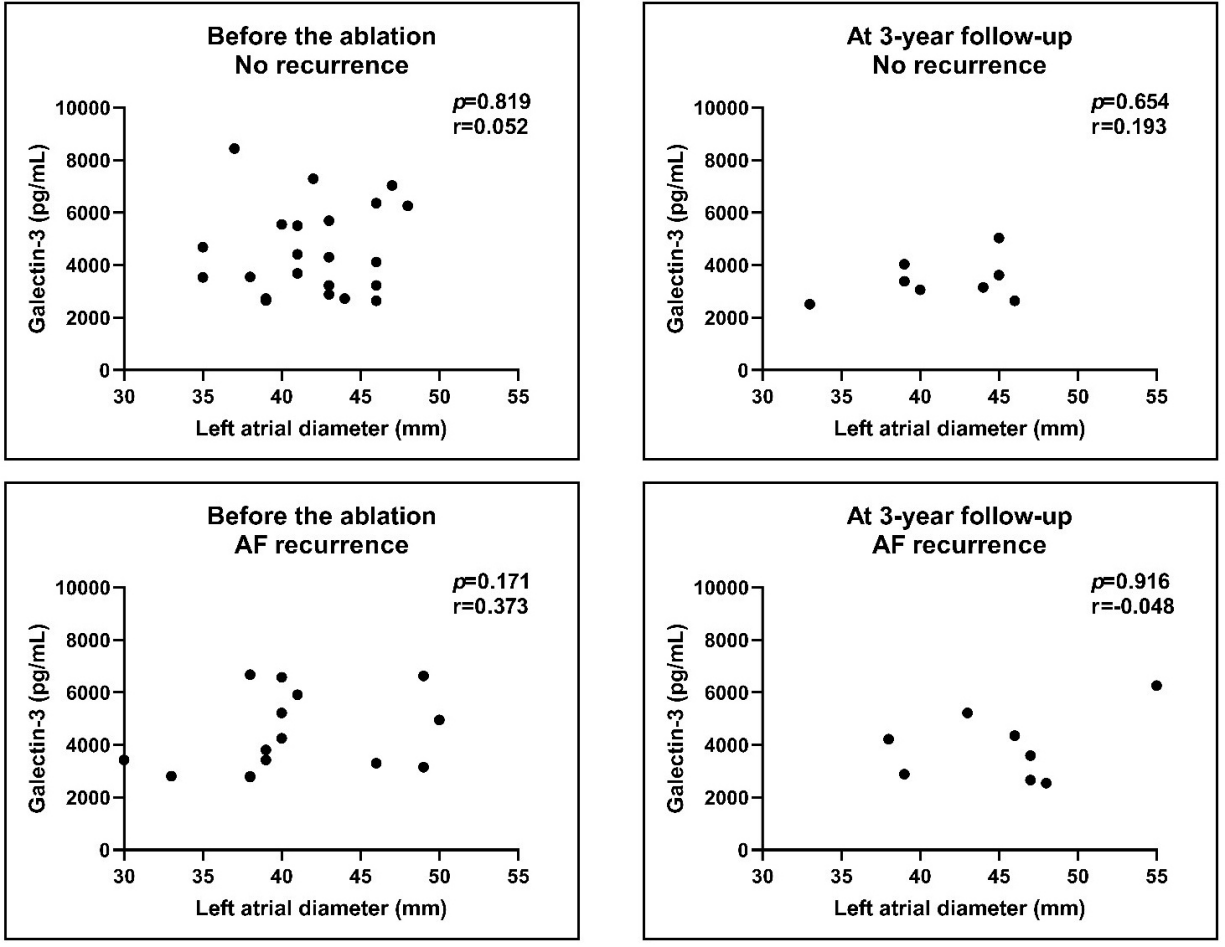
**Correlation of Galectin-3 levels with LA diameters before and after the procedure**. We compared the no recurrence group with the AF recurrence group. There was no correlation between the pre-ablation and post-ablation levels of Galectin-3 with the LA diameters. Results of Spearman’s correlations. AF, atrial fibrillation; LA, left atrial.

**Fig. 7.**
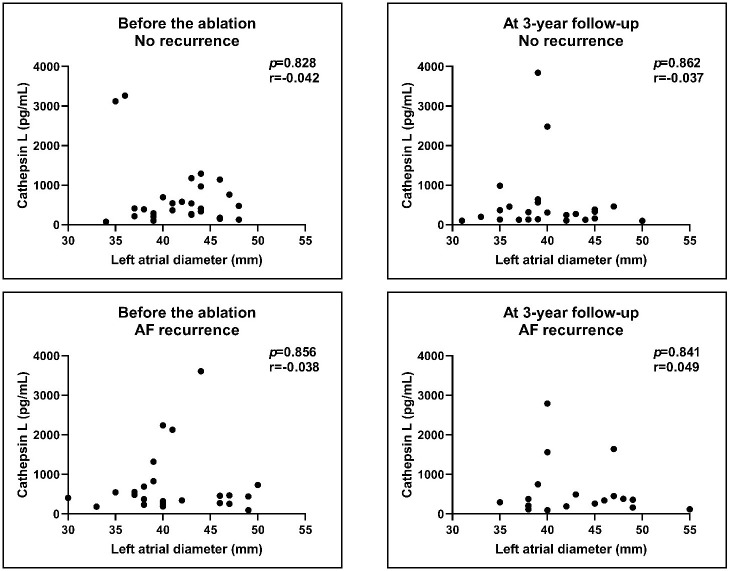
**Correlation of Cathepsin L levels with LA diameters before and after the procedure**. We compared the no recurrence group with the AF recurrence group. There was no correlation between the pre-ablation and post-ablation levels of Cathepsin L with the LA diameters. Results of Spearman’s correlations. AF, atrial fibrillation; LA, left atrial.

We found positive correlation between Caspase-3 levels and LA diameters in the AF recurrence group both before (r = 0.477; *p *= 0.018) and after the procedure (r = 0.533; *p *= 0.019; Fig. 4). There was no correlation between levels of the other biomarkers and LA diameters either pre- or post-ablation (Figs. 5,6,7).

## 4. Discussion

### 4.1 Main Findings

A 3-year clinical success after the successful acute isolation of all PVs with any of 3 different ablation technologies was demonstrated in 37 out of 63 patients (58.7%) with female sex being the only significant difference between the two groups. None of the biomarkers measured at baseline were associated with the arrhythmia outcome in our study. Levels of CA-125 demonstrated no significant change before versus 3 years after AF ablation. On the contrary, all the other biomarkers (Galectin-3, Cathepsin-L and Caspase-3) demonstrated a significant decline in all patients regardless of the arrhythmia outcome during long-term follow-up. The mean diameters of the LA and LVEF demonstrated no statistically significant change in any group. Positive correlation was found between Caspase-3 levels and LA diameters in the AF recurrence group both before and after the procedure.

### 4.2 Our Results in the Context of Published Data

The serum/plasma concentrations of these potential biomarkers in patients with versus without AF have been investigated by several studies.

CA-125 is a known tumor marker for the neoplasm of the ovarium and other organs. CA-125 has also been demonstrated strong predictor of mortality in patients with decompensated HF. Dudink *et al*. [5] investigated over 90 cardiovascular blood biomarkers in 60 AF patients without overt forms of cardiovascular diseases, and compared with 120 matched individuals without known AF. Higher CA-125 levels were demonstrated to be independent predictors of idiopathic AF. In a meta-analysis [6], 7 out of 9 studies reported significantly higher CA-125 levels in patients with AF as compared with those in SR using a cut-off level at 35 U/L. These studies included heterogeneous patient cohorts with significant comorbidities like HF and inflammatory conditions. Neither Dudink’s nor Cheung’s report contained data on the relationship between CA-125 and AF ablation results or on the long-term changes in CA-125 levels. In our study including patients free of clinical and echocardiographic signs of HF, the levels of CA-125 were lower (10 U/L before the ablation and 9 U/L at the end of follow-up) than the 35 U/L cut-off proposed by this meta-analysis and no statistical difference was demonstrated between our patients with versus without AF recurrence. Of note, CA-125 was the only biomarker among the 4 substances we studied which demonstrated no change during a 3-year follow-up. In context with these published data, our results may suggest, that CA-125 concentrations may rather be related to HF or other comorbidities with significant inflammatory response than to AF *per se* and may not be used to predict ablation success in patients with idiopathic AF.

Very limited human data have been reported on *Cathepsin-L* as a biomarker of cardiovascular diseases. Mehra *et al*. [7] described a correlation between the increased expression of Cathepsin-L in peripheral blood mononuclear cells with the severity of left ventricular dysfunction in patients with dilated cardiomyopathy. In the investigation of Dudink *et al*. [5], Cathepsin L concentrations were higher in AF patients as compared with controls in SR; however, this correlation was not found to be an independent predictor of the arrhythmia. The expression of* Caspase-3 *was investigated by Chen *et al*. [8] in patients with rheumatic heart disease undergoing valve replacement. Caspase-3 expression was increased in permanent atrial fibrillation as compared with the expression in SR. Further, Caspase-3 levels demonstrated a positive correlation with both LA dimensions and AF durations. In our study, the baseline levels of neither Cathepsin L nor Caspase-3 demonstrated an association with AF recurrence. However, a significant decrease in the concentrations of both substances was demonstrated at the end of the 3-year follow-up after the ablation. In addition, Caspase-3 was the only biomarker in our study which demonstrated a positive correlation with LA diameters in the AF recurrence group. Of note, 23% of our patients with recurrence had persistent AF as compared to only 8% in the no recurrence group. Although Caspase-3 levels were not associated with AF recurrence in our investigation, the most significant decrease after the ablation was demonstrated with this substance. Based on these observations it might be speculated that Caspase-3 levels are sensitive markers of fibrosis mainly in patients with more persistent forms of AF or with high AF burden.

Higher *Galectin-3* levels were consistently demonstrated in patients with versus without AF [9, 10]. The majority of the patients in these studies had persistent or long-standing persistent AF. Furthermore, Galectin-3 has also been tested as a potential predictor of AF recurrence after AF ablation. Based on the measurements in 160 patients (55% had paroxysmal AF; mean LA diameter 42 mm) before and 12 months after a successful PVI. Clementy *et al*. [11] reported a 1-year arrhythmia-free survival rate of 91% in those with Galectin-3 level <15000 pg/mL and LA diameter <40 mm. The predictive value of Galectin-3 for AF recurrence demonstrated in this, but not in our study could be explained by the longer follow-up in our examination as well as the differences in baseline patient characteristics. Patients with recurrence were older (64 ± 10 years vs 59.9 ± 8.5 years), had larger LA (45 mm vs 41 mm) and more comorbidities including HF (33% vs none) and higher proportion of patients with persistent AF (64% vs 23%) in the report of Clementy [11] versus in our cohort, respectively. Again, these differences might suggest that Galectin-3 concentrations may rather be related to HF or other comorbidities as well as to more persistent forms of AF. This speculation is further supported by another investigation from the same group [12] including patients with persistent AF and systolic HF (LVEF <40%), who underwent PVI. In this study the mean LA volume was 48 ± 16 mL/$m^{2}$ and over 21,000 pg/mL serum Galectin-3 levels were measured. In another study [13] including patients with long standing persistent AF, enlarged LA (mean LA diameter: 47.8 ± 1.4 mm) and structural heart disease higher Galectin-3 levels were measured in blood samples obtained from the LA cavity and from the coronary sinus in patients with persistent, than in those with paroxysmal AF. In addition, Galectin-3 levels >13,050 pg/mL in CS and >11,900 pg/mL in LA were predictive of recurrences after a single ablation. Importantly, markedly lower levels of Galectin-3 were measured (4209 pg/mL in patients with no recurrence and 3810 pg/mL in the AF recurrence cohort) in our study.

Based on our results in view of published data, markers of atrial fibrosis may predict recurrence after AF ablation in patients with more advanced state of LA disease, mostly in those with significant comorbidities and longer-term AF duration but not with a structurally normal heart, normal size LA and mainly paroxysmal form of the arrhythmia. Lower initial concentrations of biomarkers, especially of Galectin-3 measured in our cohort are in line with this explanation. The shorter follow-up durations and the uncertainties in the evaluation of recurrence in the absence of continuous long-term monitoring provide further explanations of the discrepancies between our results and the findings of other investigators.

With the exception of CA-125, all the other biomarkers included in our research demonstrated a significant decline in their concentration by the end of the 3-year follow-up. This is a novel observation, as no data have been reported on long-term changes after AF ablation; therefore, our findings await confirmation by others. A possible explanation we propose is that AF ablation might reverse fibrotic alterations by reducing the AF burden post-ablation even in those patients who have AF recurrence. Recent studies demonstrated a significant decrease in AF burden in patients with recurrence, which possibly explains the significant improvement in outcome especially in HF patients [17], an observation leading to a paradigm shift in the assessment of the long-term clinical value of AF ablation. Our findings might provide further evidence supporting this concept. Prospective studies including continuous monitoring and serial measurements of fibrosis markers could provide solid evidence to answer these questions.

## 5. Conclusions

Our results demonstrated that the levels of CA-125, Caspase-3, Cathepsin L and Galectin-3 were not associated with AF recurrence after PVI in patients with a structurally normal heart and mainly paroxysmal AF. Except for CA-125, all the other biomarkers demonstrated a significant decrease during a 3-year follow-up post-ablation. Additionally, Caspase-3 levels demonstrated a positive correlation with LA dimensions in patients with AF recurrence.

## 6. Limitations

This was a prospective single-center study including a limited number of patients. Our cohort consisted of patients with a structurally normal heart and mostly paroxysmal AF, thereby, our results may not apply to patients with significant comorbidities and more persistent forms of AF. Arrhythmia evaluation during follow-up was based on patient symptoms and on regular but not continuous arrhythmia monitoring, which might pose uncertainties regarding the precise capture of all recurrences.

## Data Availability

The data sets generated and analyzed in the current study are not publicly available due to institutional policies, but are available from the corresponding author on reasonable request.
